# The Role of Response-Shift in Studies Assessing Quality of Life Outcomes Among Cancer Patients: A Systematic Review

**DOI:** 10.3389/fonc.2019.00783

**Published:** 2019-08-20

**Authors:** Gabriela Ilie, Jillian Bradfield, Louise Moodie, Tarek Lawen, Alzena Ilie, Zeina Lawen, Chloe Blackman, Ryan Gainer, Robert D. H. Rutledge

**Affiliations:** ^1^Department of Community Health and Epidemiology, Faculty of Medicine, Dalhousie University, Halifax Regional Municipality, NS, Canada; ^2^Department of Urology, Faculty of Medicine, Dalhousie University, Halifax Regional Municipality, NS, Canada; ^3^Department of Psychology and Neuroscience, Dalhousie University, Halifax Regional Municipality, NS, Canada; ^4^Department of Radiation Oncology, Faculty of Medicine, Dalhousie University, Halifax Regional Municipality, NS, Canada; ^5^Urology, Halifax Infirmary—QEII—Nova Scotia Health Authority, Halifax Regional Municipality, NS, Canada; ^6^Nova Scotia Cancer Centre, Queen Elizabeth II Health Sciences Centre, Halifax Regional Municipality, NS, Canada

**Keywords:** cancer, survivorship, cancer patients, response-shift, quality of life, patient reported outcomes, measurement of oncological outcomes, health measurement

## Abstract

**Objective:** Response-shift has been cited as an important measurement consideration when assessing patient reported quality of life (QoL) outcomes over time among patients with severe chronic conditions. Here we report the results of a systematic review of response shift in studies assessing QoL among cancer patients.

**Methods:** A systematic review using MEDLINE, EMBASE, and PsychINFO along with a manual search of the cited references of the articles selected, was conducted. A quality review was performed using STROBE criteria and reported according to PRISMA guidelines.

**Results:** A systematic review of 1,487 records published between 1,887 and December 2018 revealed 104 potentially eligible studies, and 35 studies met inclusion criteria for content and quality. The most common cancer patient populations investigated in these studies were breast (18 studies), lung (14 studies), prostate (eight studies), and colorectal (eight studies). Response shift was identified among 34 of the 35 studies reviewed. Effect sizes were reported in 17 studies assessing QoL outcomes among cancer patients; 12 of which had negligible to small effect sizes, four reported medium effect sizes which were related to physical, global QoL, pain, and social (role) functioning and one reported a large effect size (fatigue). The most prevalent method for assessing response shift was the *then-test*, which is prone to recall bias, followed by the *pre-test and post-test* method. Given the heterogeneity among the characteristics of the samples and designs reviewed, as well as the overall small to negligible effect sizes for the effects reported, conclusions stating that changes due to internal cognitive shifts in perceived QoL should account for changes observed in cancer patients' QoL outcomes should be interpreted with caution.

**Conclusion:** Further work is needed in this area of research. Future studies should control for patient characteristics, time elapsed between diagnosis and baseline assessment and evaluate their contribution to the presence of response shift. Time between assessments should include short and longer periods between assessments and evaluate whether the presence of response shift holds over time. Possible avenues for inquiry for future investigation are discussed.

## Introducion

Measurement change in patient reported quality of life (QoL) outcomes is an urgent necessity of clinical practice. Response-shift refers to measurement of patient reported outcomes that reflect better outcomes over time not because the patient is doing better but because the patient has now adapted, psychologically, to match their new life circumstances (e.g., urinary incontinence), in order to better cope with them (1, 2). This particular “shift” in an individual's response is considered to involve a re-prioritization of values (e.g., physical function is valued less than cognitive function whereas prior to diagnosis their priority may have been reversed), a recalibration (e.g., “I will survive this, even if the quality of my life will significantly change”), or reconceptualization (e.g., significantly changing standards for interpreting meaning; what constitutes “good” now becomes different than a recent previously held belief) (2). In 1999, Albrecht and Devlieger used the term “disability paradox” to describe the notion that people with disabilities report to experience a much better QoL than expected and this concept has become a key component of response shift (3). Some cancer patients experience large amounts of pain or side-effects due to their condition or treatment such as surgery, chemotherapy or radiation therapy (2, 4). The distress associated with the diagnosis often forces patients to engage in cognitive reframing of their circumstances to ease the psychological pain they are experiencing (4, 5). This process includes a *re-prioritization* of previously held values, internal standards, and expectations in order to help the individual cope with high levels of pain (2, 6). Taking these changes into account when assessing QoL among cancer patients during the diagnosis-to-survival continuum, however, is both important and challenging. Measurement of patient reported outcomes assumes relatively good within-individual stability and consistency in ratings (6). This assumption translates to feedback for health professionals with respect to how treatments and interventions affect patients. If large error variations exist between patients' responses due, not to external circumstances but rather, to changes in internal standards and reconceptualization, then these patient reported outcomes lose the predictive value they are attributed. A meta-analysis reported in 2006 showed statistically significant response-shift among most of the studies identified (7). However, effect sizes associated with response-shift effects were small, whereby the largest ones were reported for fatigue and global health related quality of life (QoL) (7). Patient reported outcomes are particularly important in cancer research aimed at identifying treatment side effects. These outcomes help to inform patients and clinicians in the treatment decision-making process at the start of the cancer journey, as well as in the development of establishing standards of patient care and interventions aimed at improving patients' QoL. Thus, the cancer population is a particularly clinically–relevant subgroup to examine with regard to the presence or absence of response-shift.

Response shift has been commonly measured in three ways. Using the *pre-test/post-test method*, patients complete a baseline assessment (pre-test) and then they complete an identical assessment after a period of time (post-test) (7–9). In oncology research, the post-test is usually administered after the cancer treatment (2). The pre-test/post-test design is easy to administer to patients but requires large samples for analysis and is difficult to interpret. Changes from pre- to post-test could be representative of a response-shift, QoL changes due to treatment, or both. The *then-test method* is the second most commonly used method for assessing response-shift and consists of adding one extra step to the pre/post-test, administered at the same time as the post-test. During this additional (then) test, the patient is asked to rate their QoL outcomes retrospectively, thinking of the pre-test time, but using their current value judgments and perceptions (9). Response shift is calculated as the difference between the then- and pre- tests, while true changes in QoL are calculated as the difference between the post- and then tests (1, 9). The then-test is easy to analyse and interpret, however it is susceptible to recall bias and is more burdensome due to the addition of one extra (then) test (9). Finally, in the *anchor/ideal scale design*, patients are asked to state their ideal response to a question or to provide an upper and lower limit (i.e., anchors) of a specific domain at both the pre-test and post-test (9). Changes between the pre-test and post-test of either the ideal or anchors indicate a recalibration response shift (1, 7, 9). This design type can be easily analyzed and interpreted, but it is susceptible to ceiling effects and does not properly measure reconceptualization and reprioritization (3, 9, 10).

One of the major goals of assessing quality of life changes over time is to discern to what extent changes reported over time represent changes that have to do with the clinical intervention/treatment and to what extent they reflect confounds and measurement error (factors that are not accounted for but that exert influence on the outcomes, including response shift). It is usually assumed that patients' internal states are more or less stable over time (regression to mean), thus patient reported outcomes are meaningful predictors of patient outcomes (2). If for any number of reasons, the person's perception of the construct under evaluation changes over time, then comparison of the two or more longitudinal assessments during the cancer journey (e.g., diagnosis, during treatment, post-treatment) may be distorted and lead to the development of unnecessary interventions. If changes in internal states affect patient reported outcomes by means of response shift, then these changes should be accounted for in evaluations of patient reported outcomes to fine-tune the measurement process and arrive at accurate assessments that lead to reliable patient interventions (1, 2, 9, 11–13). If response shift is a significant predictor of QoL outcomes, its effect size will have important implications for assessing the effect of cancer treatments on patient reported QoL as results may reflect a response shift, a treatment effect, or a complex combination of both (7, 10, 13). Clarifying these contributions to QoL measurement may help explain paradoxical findings in the literature and provide further insight into the discrepancies between clinical measures of health and patients' own evaluations of their health. Additionally, knowledge of response shift and its measurement would lead to design adjustments for the sensitive assessment of QoL longitudinal data, ultimately leading to improved interventions that positively impact patients' lives (2, 9, 14).

To our knowledge, only one review and one meta-analysis on the evaluation of response shift have been previously conducted and none were exclusively evaluative of cancer populations (7, 12). The 2006 meta-analysis examined the presence of response shift in studies assessing all forms of chronic conditions (7), while the 2011 review examined the presence of response shift exclusively in prostate cancer studies (12). This is the first systematic review of response shift that focuses exclusively on cancer studies. The aims of this study are to review the evidence of response shift in studies assessing the QoL of cancer patients by way of examining the methods utilized to assess response shift, the QoL domains assessed and found to be prone to response shift, the length of time between assessments, and types of patient characteristics and external factors that may have contributed to the emergence of a response-shift in these studies.

## Methods

A systematic search of English-language literature using MEDLINE (1946-April 2017), EMBASE (1974-April 2017), and PsychINFO (1887-April 2017) was performed and a total of 1,365 possible articles were obtained, evaluating the presence of response-shift in cancer patients populations where quality of life outcomes were assessed. A manual search of the cited references of the selected articles did not result in additional articles. A second search of articles from April 2017- December 2018 was performed December 2018 using the exact same databases and search terms, resulting in an additional 122 possible articles for a total of 1,487 records identified through database searching. Appendix 1 lists the search strategy performed on MEDLINE as an example of the literature search performed in each database. The search words used to obtain these articles included *neoplasms* (exploded), cancer^*^, carcinoma^*^, malignan^*^, tumor^*^, neoplas^*^, adeno^*^, matasta^*^ (terms combined using an OR statement), followed by response adj (shift^*^ or change^*^), recalibrat^*^, reprioritiz^*^ or reprioritis^*^, reconceptualiz^*^ or reconceptualis^*^ (terms combined using an OR statement). Articles of interest included quantitative studies (observational studies, cohort studies, case-control studies, cross-sectional studies) that directly assessed patients of any gender, age, or cancer type on response shift and QoL. Articles without primary data (commentary, letters, reviews, editorials, and methods papers), and dissertations were excluded. Articles that assessed the impact of an intervention on QoL were also excluded.

Information was extracted primarily from the “Results,” “Discussion,” and “Methods” sections with some input from the “Background” section. Extracted information included study characteristics, type of method used to assess response shift, participant characteristics and whether they were assessed in the evaluation of the response shift effects, type and localization of cancer, severity of cancer, time between diagnosis and treatment, time elapsed between assessments, methods and results pertaining to response shift, types of QoL outcomes and an indication as to whether a response shift effect was observed, and the authors' interpretation of results and conclusions. Internal validity was evaluated by examining the study design (blinding, statistical tests, reliability, participant recruitment, study limitations, validity, and biases) and external validity was based on whether or not the sample was representative of the entire population. Effect sizes were evaluated using Cohen's criterion for significance based on differences in means as reported in the studies reviewed. Effect sizes *d* = < 0.5 were considered small, between 0.5 and 0.8 of moderate effect size and >0.8 were considered large.

## Results

After removal of duplicates, 999 articles remained. The electronic records were collected in a Research Information System (RIS) data file. Titles and abstracts of the electronic search results were screened by two authors (JB, LM) to identify the relevant studies. Articles that described observations of cancer patients, and discussed response shift, recalibration, reprioritization, and reconceptualization were then further assessed. One hundred and four articles were selected for full-text review. Further screening of the potentially eligible articles through full-text examination resulted in the exclusion of 69 articles and the selection of only 35 of the remaining articles for final inclusion.

Characteristics for data extraction included study characteristics, sample characteristics, demographics, response shift predictors, QoL outcomes, age at diagnosis, cancer type and localization of cancer, treatment type, and time elapsed between treatment and diagnosis. There were no limitations to the population size, age, or gender.

**Figure 1:**
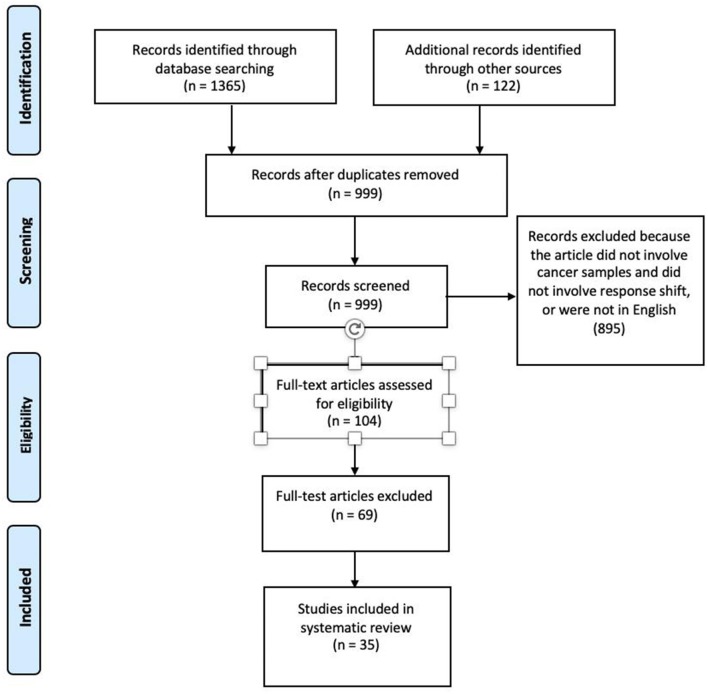
PRISMA diagram depicting the flow of information through the different phases of the systematic review.

Two authors (JB and LM) independently evaluated the relevance and quality of the articles in the search and extracted data using data abstraction forms. The STROBE (Strengthening the Reporting of Observational Studies in Epidemiology) criteria for quality assessment and the Ottawa-Newcastle Quality Assessment Scale were applied to evaluate each article on study quality and external and internal validity (15). Agreement between the two raters was very high (Cohen's kappa = 0.86). Results are reported according to the PRISMA (Preferred Reporting Items for Systematic Reviews and Meta-Analyses) guidelines (16). A PRISMA flowchart, shown in Figure 1, was created to demonstrate the number of articles at each stage of data acquisition and the number of articles that were excluded at each stage.

Among the 35 studies included, all were published in the year 2000 or later, with the exception of Sprangers et al. published in 1999 (17). Table 1 displays the study characteristics. The majority of the studies were conducted in the Netherlands (4, 17, 21, 24, 26, 32, 34–36, 43–46, 48), followed by France (19, 23, 27, 37, 39, 41), and Germany (25, 28, 29, 47). Other countries included the United States of America (18), Switzerland (20, 42), Sweden (22, 33), Norway (14, 31), Ireland (38), Japan (30), and Australia (13, 40). Four studies were case-controls (28, 29, 33, 38), while the remaining 31 were unmatched cohorts. There was a large range of QoL measures used, with EORTC-QLQ-C30 being the most prevalent (14, 17, 19, 22, 24, 25, 27, 37, 39, 41, 43, 48). Twenty-two of the studies looked at global/general/overall QoL results (13, 14, 17, 19–24, 26–32, 34–37, 44, 46), and all studies measured one or more individual aspects of QoL. Response shift was assessed in nine specific aspects of QoL: physical functioning (21 studies) (14, 19–23, 25–27, 30, 32, 34–37, 41–44, 46, 47), role functioning (the capacity of an individual to perform activities typical to specific age and particular social responsibility; assessed in 11 of the 35 studies reviewed) (19, 23, 25, 27, 30, 34, 36, 37, 41, 44, 46), emotional functioning (10 studies) (19, 21, 23, 25–27, 30, 37, 41, 44), cognitive functioning (eight studies) (17, 19, 21, 23, 25, 27, 37, 41), sexual functioning (five studies) (19, 23, 27, 35, 37), social functioning (12 studies) (19, 21, 23, 25, 27, 30, 36, 37, 41–44), fatigue (16 studies) (14, 17–19, 23, 24, 27, 30, 32, 36, 37, 42, 43, 45, 46, 48), pain (11 studies) (4, 14, 19, 23, 27, 30, 34, 37, 43, 44, 46), and mental health (15 studies) (22, 28, 29, 33–36, 38–40, 42–44, 46, 47). Aside from aspects of QoL, 13 studies examined other outcomes (19, 23, 24, 26, 27, 34, 37, 41–44, 46, 47), such as communication, relationship with physician, and dyadic congruency. The duration of follow-up tests from baseline ranged from 6 days to 2 years, with most studies assessing response shift at 3 or 6 months post-diagnosis, and having no more than 6 months elapsed between pre to post-test. Ito et al. had the smallest sample size (*N* = 18, *n* = 13 at follow up) (30) and Verdam et al. had the largest sample size (*N* = 1,157, 1,029 at follow up) (43) at baseline. Mean sample size at first follow-up, for the 35 studies was 0.203 participants (*SD* = 190.68), and the median was 170 participants. Twelve of the 35 studies reviewed had <100 participants enrolled at first follow-up.

**Table 1:** Characteristics of the studies reviewed, *n* = 35.

**References**	**Design**	**Country**	**Cancer type**	**QoL measure**	**Aspect of QoL assessed**	**Other outcomes**	**Sample size**	**Limitations**	**Time between assessments**
Andrykowski et al. (18)	Cohort	US	Breast	FSI; FSI-TT; FCS	Fatigue	None	*N* = 102 at pre-treatment; *N* = 73 at T1; *N* = 39 at T2	(1) 62% attrition from baseline to post-treatment testing. (2) Sample lacks racial/ethnic representation.	Pre-treatment to post-treatment 1: M = 70.2 days (*SD* = 40.9; range 22–202) Pre-treatment to Post-treatment 2: M = 193.8 days (*SD* = 57.3; range 115–364)
Anota et al. (19)	Cohort	France	Breast	EORTC QLQ-C30; EORTC QLQ-BR23 BC	Global QoL; physical, emotional cognitive, role, and social functioning, fatigue, pain, sexual functioning	Nausea and vomiting; dyspnea; insomnia; appetite loss; constipation; diarrhea; financial difficulties; body image; future perspectives; systemic therapy side effects; arm and breast symptoms; hair loss	*N* = 317 at baseline; *N* = 311 at T1; *N* = 304 at T2; *N* = 290 at T3	(1) Potential for recall bias using “Then” test. (2) Failure to report sample demographic.	Baseline to T1 (discharge following initial hospitalization): median = 6 days, (range 1.5–81.5) Baseline to T2: 3 months Baseline to T3: 6 months
Bernhard et al. (20)	Cohort	Switzerland	Colon	LASA scale anchored at ‘perfect health-worst health'	Global QoL; physical functioning, subjective health estimation	None	*N* = 187 recruited; *N* = 130 completed pre- and post-tests	(1) Duration between T1 (baseline/pre-test) and T2 (surgery) was too short.	Baseline/ “pre”-test to surgery: “Then”-test median = 12 days Adjuvant therapy: Baseline/”pre”-test to “then”-test: median = 50 days; “Then”-test to “post”-test: median = 14 days
Brinksma et al. (21)	Cohort	Netherlands	Multiple types: hematologic (*N* = 12); brain tumor (N * =* 7); solid tumor (*N* = 18)	PedsQL, Cantril's ladder, PPS, MSAS	Physical, emotional, cognitive, and social functioning, overall QoL		Child report: *N* = 51 enrolled, *N* = 37 completed all measures; Parent report: *N* = 100 enrolled, *N* = 80 completed all measures	(1) Small sample size (2) Broad distribution of age (3) 27% children and 20% parents, attrition, respectively	Pretest 2 weeks after diagnosis, post-test and “Then” test 3 months later
Broberger et al. (22)	Cohort	Sweden	Lung	EORTC-QLQ-C30	Global QoL; fatigue, physical functioning	None	*N* = 126 enrolled; *N* = 115 at T1; *N* = 89 at T2	(1) Attrition of 30% by T2. (2) Possible recall bias.	T1: 3 months after baseline T2: 6 months after baseline
Dabakuyo et al. (23)	Cohort	France	Breast	QLQ-C30, BR23, and EurQOL-EQ-5D	Global QoL; physical, emotional cognitive, role, and social functioning; fatigue; pain; sexual functioning	Nausea and vomiting; dyspnea; insomnia; appetite loss; constipation; diarrhea; financial difficulties;	*N* = 381 enrolled; *N* = 320 completed all measures	(1) 17% attrition (2) Short time between tests	Baseline to the end of the 1st medical examination/ hospitalization: <15 days for 79% of sample (*N* = 301)
						body image; future perspectives; systemic therapy side effects; arm and breast symptoms; hair loss			
Echteld et al. (24)	Cohort	Netherlands	Multiple types: lung (*N* = 6); colorectal (*N* = 4); urogenital (*N* = 3); breast (*N* = 4); melanoma (*N* = 3); sarcoma (*N* = 2) other (*N* = 7)	SEI QoL-DW	Pain; fatigue; global QoL	Cues: affiliation; activities/hobbies/ interests; health progression; state of mind; quality of care; role, mental, physical functioning; religion/spirituality, harmony and acceptance; work/finances/ practical issues; autonomy/control; outlook	*N* = 29 patients selected from sample of 78 patients. *N* = 29 at T0; *N* = 16 at T1;	(1) Heterogeneity of cancer type (2) Small sample size.	Baseline: within 24 h of hospital admission T1: 7–14 days later
Gerlich et al. (25)	Cohort	Germany	Prostate	EORTC-QLQ-C30	Physical, role, emotional, cognitive, and social functioning	None	*N* = 437 recruited; *N* = 402 at follow-up	(1) Results pertain to the whole sample of patients, so unable to examine response shift on an individual level. (2) Short time-frame used, so unable to assess RS in later disease. (3) Potential bias from different contexts of both assessment occasions (hospital stay for baseline vs. at home for follow-up)—this constitutes method variance.	Baseline: start of treatment Follow-up: 3 months after baseline
Hagedoorn et al. (26)	Cohort	Netherlands	Multiple types: advanced breast (*N* = 53); gastrointestinal tumors (*N* = 30); lymphomas (*N* = 32); genitourinary (*N* = 16); lung (N = 15); gynecological (*N* = 12)	EORTC-QLQ-C30	Physical and emotional functioning, global QoL	Relative evaluation on a 7-point scale: “When you compare yourself to other people in a similar situation, how would you say you are doing?”	*N* = 307 recruited; *N* = 240 competed study	(1) Heterogeneity of cancer type (2) 22% attrition	T1: during 2nd/3rd cycle of chemotherapy T2: 3 months after T1
Hamidou et al. (27)	Cohort	France	Breast	EORTC-QLQ-C30 and BR-23	Global QoL; physical, emotional cognitive, role, and social	Nausea and vomiting; dyspnea; insomnia; appetite loss;	*N* = 359 at baseline; *N* = 357 completed at least 1 follow-up	(1) Reassessed QOL at 3 months, T3 “Then”-test not	T0: Inclusion T1: end of 1st hospitalization
					functioning, fatigue; pain, sexual functioning	constipation; diarrhea; financial difficulties; body image; future perspectives; systemic therapy side effects; arm and breast symptoms; hair loss		measuring the same construct as T1 and T2	T2: 3 months after 1st hospitalization T3: 6 months after 1st hospitalization
Hinz et al. (28)	Case-control	Germany	Multiple types: prostate (*N* = 242); kidney (*N* = 14); bladder (*N* = 14); testicles (*N* = 3); penis (*N* = 1); renal pelvis (*N* = 1)	Questionnaire on Life Satisfaction, PHQ-2, GAD-2	Anxiety; depression; distress; health satisfaction	None	*N* = 427 recruited; *N* = 275 completed all questionnaires	(1) Heterogeneity of cancer type (2) Recall bias (3) 36% attrition rate	T1: 2 days before discharge T2: 2 weeks after discharge T3: 3 months after discharge
Hinz (29)	Case-control	Germany	Breast	EQ-5D VAS, PHQ-4. LOT revised	General health	None	*N* = 338 recruited; *N* = 308 at follow-up	(1) Patients had already begun treatment (2) At least 6 months since diagnosis	Baseline: radiological follow-up examination Follow-up: 3 months later
Ito et al. (30)	Cohort	Japan	Rectal	SF-36	Mental health, role limitations due to emotional health, social functioning, vitality, general health, physical functioning, role limitations due to physical health, bodily pain	None	*N* = 18 recruited; *N* = 13 completed all questionnaires	(1) Small sample (2) Use of generic QoL measure (SF-36) instead of disease-specific scale (3) Recruitment hospital (with high level of expertise) may not have representative of other hospitals in and the typical healthcare received in Japan	Baseline: prior to surgery Follow-up: 2 months after surgery
Jakola et al. (31)	Cohort	Norway	Glioma	EQ-5D 3L, VAS	Global QoL	None	172 at baseline; *N* = 106 at follow-up; *N* = 73 completed “Then”-test	(1) Potential selection bias via exclusion of patients with terminal illness, severe cognitive impairment, or severe language problems (2) Recall bias	Baseline: 1–3 days before surgery Follow-up: 6 months after surgery
Jansen et al. (32)	Cohort	Netherlands	Breast	SF-36, RSCL	Fatigue, global QoL, psychological well-being, physical function	None	*N* = 50 at baseline; *N* = 46 at follow-up	(1) Many pairwise *t*-tests (2) 58% attrition	Not specified
Jorngarden et al. (33)	Case-control	Sweden	Multiple types: CNS tumor (*N* = 2); Ewing sarcoma (*N* = 4); leukemia (*N* = 18); lymphoma (*N* = 20); osteosarcoma (*N* = 8); other (*N* = 4)	HADS, SF- 36	Depression, anxiety, vitality, mental health	None	*N* = 56 at T1; *N* = 53 at T2; *N* = 45 at T3; *N* = 42 at T4	(1) Small sample (2) Heterogeneity of cancer type	T1: shortly after diagnosis T2: 6 months after diagnosis T3: 12 months after diagnosis T4: 18 months after diagnosis
King-Kallimanis et al. (34)	Cohort	Netherlands	Multiple types: lung (*N* = 36); pancreatic (*N* = 49); esophageal (*N* = 55); cervical (*N* = 62)	SF-36	Physical functioning, role functioning, pain, social functioning, mental health, vitality, general QoL	Health status, optimism, upward comparison	*N* = 202 recruited; attrition from baseline to 3 months follow-up not specified	(1) Heterogeneity of cancer type	Baseline: before surgery Follow-up: 3 months after surgery
Korfage et al. (35)	Cohort	Netherlands	Prostate	SF-36, EQ-5D VAS	Mental health, global QoL, physical function, sexual function	Vitality	*N* = 52 at baseline; *N* = 51 at follow-up	(1) Small sample (2) Used different modes of questionnaire administration at baseline and follow-up (3) Lack of information on marital status and education	Baseline: 1-month post-diagnosis (before treatment) Follow-up: 7 months post-diagnosis
Kvam et al. (14)	Cohort	Norway	Multiple myeloma	EORTC-QLQ-C30	Pain, fatigue, global QoL, physical function	None	*N* = 260 recruited; *N* = 239 completed all questionnaires	(1) Lack of demographics information on participants	T1: Baseline T2: 3 months after T1
Oort et al. (36)	Cohort	Netherlands	Multiple types: lung (*N* = 29); pancreas (*N* = 43); esophageal (*N* = 46); cervical (*N* = 52)	SF-36, MFI	Physical functioning, role functioning, pain, global QoL social functioning, mental health, fatigue	Vitality	*N* = 170 recruited; attrition from baseline to 3 months follow up not specified	(1) Heterogeneity of cancer type (2) Subjective decisions were involved in the equation modeling approach employed for the detection of response shift	Baseline: prior to surgery Follow-up: 3 months after surgery
Ousmen et al. (37)	Cohort	France	Breast	EORTC-QLQ-C30, QLQ-BR23	Global QoL, physical, emotional cognitive, role, and social functioning, fatigue; pain, sexual functioning	Nausea and vomiting; dyspnea; insomnia; appetite loss; constipation; diarrhea; financial difficulties; body image; future perspectives; systemic therapy side effects; arm and breast symptoms; hair loss	*N* = 381 recruited. QLQ-C30: *N* = 359 at T0; *N* = 340 at T1; *N* = 322 at T2. QLQ-BR23: *N* = 352 at T0; *N* = 340 at T1; *N* = 322 at T2	(1) Not all patients had confirmed breast cancer (2) Possible recall bias	T0: time of diagnosis T1: 3 months after surgery T2: 6 months after surgery
Rees et al. (38)	Case-control	Ireland	Prostate	IPSS, SPI	Urinary function	None	*N* = 76 at baseline; *N* = 74 at follow-up	(1) Uneven case to control matching	Baseline: newly diagnosed and before treatment Follow-ups: 3 and 6 months after diagnosis
Salmon et al. (39)	Cohort	France	Breast	MFI-20, QLQ-C30, State-Trait Anxiety Inventory, LOT	Fatigue, Anxiety	Optimism	*N* = 466; attrition from baseline to study completion not specified	(1) Does not report attrition, if any	Baseline: newly diagnosed Follow-ups: 10 visits over 24 months
Sharpe et al. (13)	Cohort	Australia	Multiple types: lung and colorectal primarily	SEIQoL-DW, FACT-G	Global QoL	Most important aspects of life	*N* = 56 at baseline; *N* = 28 completed all assessments	(1) 50% attrition (2) Small sample size.	T1: within 3 months of metastases diagnosis T2: 3 months after T1 T3: 6 months after T1
Sharpley and Christie (40)	Cohort	Australia	Breast	SAS, SDS	Depression, anxiety	None	*N* = 445 invited to complete survey; *N* = 197 completed survey	(1) Then-test and now-test at same time (2) 56% attrition.	Then-test and now-test at same time
Sprangers et al. (17)	Cohort	Netherlands	Multiple types: breast (*N* = 59); lung (*N* = 28); prostate (*N* = 16); Hodgkin (*N* = 2)	EORTC-QLQ-C30, MFI-20	Cognitive function, fatigue	None	*N* = 127 asked to participate; *N* = 105 at baseline; *N* = 99 at follow-up	(1) Heterogeneity of cancer type (2) 22% attrition.	Baseline: prior to radiotherapy Follow-up: after completion of radiotherapy
Tessier et al. (41)	Cohort	France	Breast	EORTC-QLQ-C30, SWLS	Physical, role, emotional, cognitive, social functioning	Life satisfaction	*N* = 215 recruited; *N* = 207 at T1 (1 year); *N* = 200 at T2 (2 years)	(1) Did not control for influence of personality traits that are known determinants of SWB and HRQoL. (2) Health utility index used does not consider pain scale. (3) No control group used for comparison. (4) RS only detected at last assessment point, so cannot determine whether phenomenon is transient	Baseline: within 1 month of breast cancer diagnosis Follow-ups: 1 and 2 years after diagnosis
Traa et al. (42)	Cohort	Switzerland	Colorectal	WHOQOL-BREF	Physical functioning; mental health; social functioning	Environment	*N* = 164 recruited; *N* = 123 at 6 months	(1) Patient attrition	T0: preoperative T1: 3 months postoperative T2: 6 months postoperative
Verdam et al. (43)	Cohort	Netherlands	Multiple types: breast (*N* = 451); prostate (*n* = 267); lung (*n* = 287); other (*n* = 152)	EORTC-QLQ-C30, EQ-5D, RSCL	Physical functioning; fatigue; social functioning; mental health; listlessness; pain	Treatment-related symptoms; sickness	*N* = 1,157 recruited; *N* = 1,029 completed the assessments	(1) 11% attrition (2) Heterogeneity of cancer type	T0: before treatment T1 to T12: every week after treatment T13 to T24: monthly for up to 2 years
Verdam et al. (44)	Cohort	Netherlands	Multiple types: breast (*N* = 158); colorectal (*N* = 105); lung (*N* = 130); other (*N* = 44)	SF-36	Mental health, role functioning, emotional functioning, physical functioning, social functioning, global QoL, physical functioning, pain	Vitality, health comparisons	*N* = 485 recruited; *N* = 447 at follow-up	(1) 8% attrition (2) Heterogeneity of cancer type	T0: prior to starting treatment T1: 4 weeks after start of treatment T2: 4 months after start of treatment Data analysis only included T0 and T1 data.
Visser et al. (45)	Cohort	Netherlands	Multiple types: head and neck (*N* = 12); gastrointestinal (*N* = 11); gynecological (*N* = 25); lung (*N* = 20); breast (*N* = 35); genitourinary tract (*N* = 76); hematological malignancies (*N* = 15); other (*N* = 5)	One-item numerical rating scale from 0 to 10	Fatigue	None	*N* = 250 recruited; *N* = 199 completed all assessments	(1) Poor measurement	Pre-treatment: approximately 2 weeks before radiotherapy Post-treatment: 2–4 weeks after completion of radiotherapy
Visser et al. (46)	Cohort	Netherlands	Multiple types: lung (*N* = 29); periampullary (*N* = 43); esophageal (*N* = 46); cervical (*N* = 52)	SF-36, MFI	Mental health, role functioning, emotional functioning, social functioning, global QoL, physical functioning, pain, fatigue	Vitality	*N* = 170; attrition from baseline to study completion not specified	(1) Heterogeneity of cancer type	Baseline: prior to surgery Follow-up: 3 months after surgery
Visser et al. (4)	Cohort	Netherlands	Multiple types: lung (*N* = 36); periampullary (*N* = 49); esophageal (*N* = 55); cervical (*N* = 62)	SF-36	Pain	None	*N* = 202; attrition from baseline to study completion not specified	(1) Heterogeneity of cancer type (2) Method variance	Baseline: prior to surgery Follow-up: 3 months after surgery
von Blanckenburg et al. (47)	Cohort	Germany	Multiple types: breast (*N* = 26); lung (*N* = 17); urologic (*N* = 14); digestive (*N* = 12); gynecologic (*N* = 8); other (*N* = 9)	Global QoL—WHOQOL-BREF−2 items; life goals questionnaire (GOALS)-−24 items; EORT-QLQ−5 items physical functioning	Global QoL, physical functioning, mental health	Life goals (affiliation, altruism, intimacy, achievement, power and variation).	*N* = 86 recruited; *N* = 44 at follow-up	(1) Heterogeneity of cancer type (2) Small sample size (3) 49% attrition	Follow-up: 20 months after the first (baseline) measurement
Westerman et al. (48)	Cohort	Netherlands	Lung	EORTC-QLQ-C30	Fatigue	None	*N* = 23 recruited; *N* = 16 completed all assessments	(1) Small sample	T1: start of chemotherapy T2: 4 weeks after T1 T3: 7-10 day after chemotherapy completion T4: 6 weeks later

*CNS, central nervous system; EORTC QLQ-BR23 BC, European Organization for Research and Treatment of Cancer (EORTC) quality of life questionnaire breast cancer-23; EORTC QLQ-C30, EORTC quality of life questionnaire core-30; ESAS, Edmonton Symptom Assessment Scale; EurQOL-EQ-5D/EQ-5D, Euro QoL five-dimension scale; VAS, Visual Analog Scale; FACT-G, Functional Assessment of Cancer Therapy; FCS, Fatigue Catastrophizing Scale; FSI, Fatigue Symptom Inventory; FSI-TT, Fatigue Symptom Inventory—Then test; GAD-2, Generalized Anxiety Disorder Questionnaire-2; HADS, Hospital Anxiety and Depression Scale; HR QoL, health-related quality of life; IPSS, International Prostate Symptom Score; LASA, linear analog self-assessment; M, mean; MFI, multidimensional fatigue inventory; MSAS, Memorial Symptom Assessment Scale; N, number of subjects; Peds QL, Pediatric Quality of Life Inventory; PHQ-2/4, Patient Health Questionnaire-2/4; PPS, Play Performance Scale; RAI MDS-PC, Resident Assessment Instrument for Palliative Care; RSCL, Rotterdam symptom checklist; SD: standard deviation; SEI QoL-DW, Individual Quality of Life (SEI QoL): a direct Weighing procedure for Quality of Life Domains; SF-36, 36-Item Short Form Health Survey; SPI, Symptom Problem Index; SWB, subjective well-being; SWLS, Satisfaction with Life Scale; T, time (e.g., T1, time 1); VAS, visual analog scale; WHOQOL-Bref, World Health Organization Quality of Life assessment instrument abbreviated version; SAS, Self-Rating Anxiety Scale; SDS, Self-rating Depression Scale; LOT, Life Orientation Test; MFI-20, Multidimensional Fatigue Inventory-20*.

Table 2 displays patients characteristics. Roughly half of the studies (*N* = 21) included both males and females in their sample (4, 13, 14, 17, 20–22, 24, 26, 30, 31, 33, 34, 36, 42–48), four studies encompassed only males (25, 28, 33, 35), and 10 studies consisted of only females (18, 19, 23, 27, 29, 32, 37, 39–41). Sex was assessed in response-shift analyses in four studies and was found to be a statistically significant contributor in two of the four studies assessed (Table 3). Of the studies which reported participants' age, 32 studies examined response shift in older patients (40–80 years old), and one study examined response shift among children (mean age 14). Age at diagnosis and time elapsed since diagnosis were a statistically significant contributor in 6 of the 10 studies, and one of four studies that assessed it's contribution to response shift analyses, respectively (Table 3). In 5 of the 7 studies that assessed the role of external factors in response shift analyses, five were statistically significant contributors (e.g., life domains, social comparisons, financial status) (Table 3). The majority of studies (16 studies) administered QoL assessment 3 months after baseline whereas nine studies measured 6 months after baseline. Only four, of the 35, studies assessed the time elapsed between diagnosis and first/baseline testing and controlled for it in their analyses. Breast cancer (18 studies) was the most common form of cancer assessed, with 10 studies examining only breast cancer (18, 19, 23, 27, 29, 32, 37, 39–41) and 8 studies focusing on breast plus other types of cancers (13, 17, 24, 26, 43–45, 47). The second and third most commonly examined forms of cancer were lung (14 studies, 2 of which included lung patients exclusively) (4, 13, 17, 22, 24, 26, 34, 36, 43–48), prostate (8 studies, 2 of which included prostate cancer patients only) (13, 17, 25, 28, 35, 38, 43, 47) and colorectal (8 studies, 2 of which were exclusively colorectal samples) (13, 24, 26, 30, 42, 44, 45, 47). Information on cancer stage was reported in about a third of the studies (18, 20, 23, 25, 27, 28, 30–32, 37–39, 41). Among those that did present information on cancer staging, the TNM classification—T1 to T4 and the AJCC stages−0 to 4, were reported most frequently. Eight of the 35 studies reviewed included patients whose cancer had metastasized (13, 26, 30, 37, 38, 43, 44, 47). The most reported treatments for cancer among the 35 studies included surgery (19 studies) (4, 18–20, 23, 25, 27, 28, 30–32, 34–39, 46, 47), radiotherapy (19 studies) (13, 17–19, 23, 25, 27–29, 31, 32, 35, 37–39, 42, 44, 45, 47), chemotherapy (15 studies) (13, 14, 19, 20, 23, 27–29, 33, 37, 39, 42, 44, 47, 48), and hormone therapy (10 studies) (13, 19, 23, 27–29, 35, 37–39). All of the studies included pre- and post-treatment assessments, except Hinz who recruited participants during a radiological follow-up (29). Two studies recruited children aged 2 to 17 years old (21, 38), while the rest of the studies included adult participants only. The majority of the 35 studies reviewed failed to provide information on participants, such as cancer type and stage, treatment type, age at diagnosis, presence of metastasis, and time between diagnosis and testing.

**Table 2:** Patients characteristics among the 35 studies included in this review.

**References**	**Cancer type**	**Cancer stage**	**Metastasis**	**Treatment**	**Time since diagnosis**	**Age at diagnosis**	**Age**	**Sex**
Andrykowski et al. (18)	Breast	0-IIIA	Not specified	Adjuvant therapy: chemotherapy or radiotherapy; Breast surgery: lumpectomy or astectomy	Not specified	Mean: 54.7 ± 10.6	Not specified	All female
Anota et al. (19)	Breast	Not specified	301 patients had a lymph node dissection	Chemotherapy; hormone therapy; radiotherapy; surgery (mastectomy)	Diagnosed between February 2006 and February 2008	Not specified	Mean: 58.4 ± 11	All female
Bernhard et al. (20)	Colon	pT1-4 pN>0 M0 and pT3-4 pN0 M0	Not specified	Chemotherapy; surgery	Not specified	Not specified	Median: 62 Range: 27–88	59% male
Brinksma et al. (21)	Hematologic; brain tumor; solid tumor	Not specified	Not specified	Not specified	Not specified	Not specified	Child report Mean: 14 Range: 8–17 Parent report Mean: 9 Range: 2–17	Child report: 54% female; Parent report: 55% female
Broberger et al. (22)	Lung	Not specified	Not specified	Not specified	3-month follow-up: 119 days 6-month follow-up: 219 days	Not specified	Mean: 64 ± 10 Range: 47–95	49% female
Dabakuyo et al. (23)	Breast	AJCC stage: 0–4	163 patients had a lymph node biopsy	Chemotherapy; hormone therapy; radiotherapy; surgery (mastectomy)	Not specified	Divided into two groups: <65 (younger); >65 (older)	Mean: 58.4 ± 11	All female
Echteld et al. (24)	Lung; colorectal: urogenital; breast; melanoma; sarcoma; other	Not specified	Not specified	Not specified	Not specified	Not specified	Mean: 55.3 Range: 27–80	69% female
Gerlich et al. (25)	Prostate	TNM classification: T1–T4	Not specified	Radiotherapy; surgery	Not specified	Not specified	Mean: 64.7 ± 7.1	All male
Hagedoorn et al. (26)	Breast; gastrointestinal tumors; lymphomas; genitourinary; lung; gynecological	Not specified	Yes, in 140 patients	Not specified	Not specified	Not specified	Mean: 50.5 ± 14.4 Range: 19–80	55% female
Hamidou et al. (27)	Breast	AJCC stage: 0–4	283 patients had LND	Chemotherapy; hormone therapy; radiotherapy; surgery	Not specified	Not specified	Mean: 58 ± 11.1	All female
Hinz et al. (28)	Prostate; kidney; bladder; testicles; penis; renal pelvis	I-IV	Not specified	Chemotherapy; hormone therapy; radiotherapy; surgery	Mean: 112.9 ± 294	Not specified	Mean: 63.7 ± 8.3	All male
Hinz (29)	Breast	Not specified	Not specified	Chemotherapy; radiotherapy; hormone therapy	At least 6 months, to over 5 years	Not specified	Mean: 66.1 ± 9.3	All female
Ito et al. (30)	Rectal	TNM classification: 1–4	TNM staging N (%): I-IIIb: 12 (92); IV: 1 (8)	Surgery	Not specified	Not specified	Mean: 66.9 ± 11.3	62% male
Jakola et al. (31)	Glioma	WHO grade II-IV	Not specified	Radiotherapy; surgery	Not specified	Not specified	Mean: 49 ± 15	26% female
Jansen et al. (32)	Breast	Early-stage	Not specified	Surgery (lumpectomy or mastectomy); radiotherapy	Not specified	Not specified	Mean: 55 ± 10	All female
Jorngarden et al. (33)	CNS tumor; Ewing sarcoma; leukemia; lymphoma; osteosarcoma; other	Not specified	Not specified	Chemotherapy	Not specified	Mean: 15.7; 13–15 years (*N* = 35); 16–19 years (*N* = 21)	Not specified	57% male
King-Kallimanis et al. (34)	Lung; pancreatic; esophageal; cervical	Not specified	No	Surgery	Not specified	Not specified	Mean: 57.3 ± 14.2	51.49% male
Korfage et al. (35)	Prostate	Not specified	Not specified	Active surveillance; brachytherapy; external radiotherapy; hormonal treatment; radical prostatectomy	Beginning of study	Not specified	Mean: 67.3 ± 4.4	All male
Kvam et al. (14)	Multiple myeloma	Not specified	Not specified	ASCT; MP ± Thalidomide; Thalidomide; Velcade	Not specified	Not specified	Median: 66 Range: 36–89	54% male
Oort et al. (36)	Lung; pancreas; esophageal; cervical	Not specified	Not specified	Surgery	Not specified	Not specified	Mean: 57.5 ± 14.1	51% male
Ousmen et al. (37)	Breast	AJCC stage: 0–4	301 patients had LND	Chemotherapy; hormone therapy; radiotherapy; surgery	Not specified	Not specified	Mean: 58.4 ± 11	All female
Rees et al. (38)	Prostate	TNM classification: T2–T4	Yes, distant metastases identified in 19 patients	Active surveillance; hormone therapy; radiotherapy	Not specified	Not specified	Mean: 72.8 ± 8.5	All male
Salmon et al. (39)	Breast	I-III	No	Surgery; chemotherapy; radiotherapy; hormone therapy	Not specified	Not specified	Mean: 57 ± 10.4	All female
Sharpe et al. (13)	Multiple, but primarily lung and colorectal	Not specified	Yes, all patients had metastatic cancer	Chemotherapy; radiotherapy; combination; other	Metastatic cancer in previous 3 months before the study	Not specified	Mean: 64 ± 8.6	49% male
Sharpley and Christie (40)	Breast	Not specified	Not specified	Not specified	Average: 2 years 8 months	Not specified	Mean: 58.76 Range: 26–85	All female
Sprangers et al. (17)	Breast; lung; prostate; Hodgkin	Not specified	Not specified	Radiotherapy	Not specified	Not specified	Median: 63 Range: 28–89	60% female
Tessier et al. (41)	Breast	TNM classification: I or II	No	Not specified	First survey distributed 1 month following diagnosis	Not specified	Mean: 53	All female
Traa et al. (42)	Colorectal	Not specified	Non-curatively treated metastases at baseline excluded	Chemotherapy; radiotherapy; surgery	Not specified	Not specified	Mean: 62 ± 8.6	71.2% male
Verdam et al. (43)	Breast; prostate; lung; other	Not specified	Yes, all with bone metastases from a solid tumor	Not specified	Not specified	Not specified	Mean: 64.87	46% female
Verdam et al. (44)	Breast; colorectal; lung; other	Not specified	Yes in 40% of patients	Chemotherapy; radiotherapy	Not specified	Not specified	Mean: 57 ± 12.1	41% male
Visser et al. (45)	Head and neck; gastrointestinal; gynecological; lung; breast; genitourinary tract; hematological malignancies; other	Not specified	Not specified	Radiotherapy	Not specified	Not specified	Mean: 64 ± 13	58% male
Visser et al. (36)	Lung; periampullary; esophageal; cervical	Not specified	Not specified	Surgery	Not specified	Not specified	Mean: 57.7 ± 14.1 Range: 27–83	51% male
Visser et al. (4)	Lung; periampullary; esophageal; cervical	Not specified	Not specified	Surgery	Not specified	Not specified	Mean: 57.28 ± 14.2	51% male
von Blackenburg et al. (47)	Breast; lung; urologic; digestive; Gynecologic; other	Not specified	Yes in 29 patients (33.7%)	Chemotherapy; radiotherapy; Surgery	Not specified	Not specified	Mean: 52.5 ± 8.1 Range: 31–65	53.5% female
Westerman et al. (48)	Lung	Not specified	Not specified	Chemotherapy	Not specified	Not specified	Mean: 58.7 Range: 46–72	47.8% male

*AJCC, American Joint Committee on Cancer; ASCT, autologous stem cell transportation; CNS, central nervous system; LND, lymph node dissection; MP, Melphalan and Prednisone; TNM, Tumor Node Metastasis; WHO, World Health Organization*.

**Table 3:** Demographics, social support, and clinical contributions of patient characteristics to main response-shift statistical analyses.

**References**	**Cancer type**	**Cancer stage**	**Metastasis**	**Tx**	**Time since dx**	**Age at dx**	**Age**	**Sex**	**Race**	**Other morbidity**	**Occupation**	**Marital status**	**Education**	**Social support**	**External factors**
Andrykowski et al. (18)	O	NS	O	S	O	O	NS for one group, S for other	O	O	O	O	O	NS	O	Pre-treatment FSI Rating: S; Days of Retrospection: NS; Study Site: NS
Anota et al. (19)	O	O	O	O	O	O	O	O	O	O	O	O	O	O	O
Bernhard et al. (20)	O	O	NS	NS	O	O	O	NS	O	O	O	O	O	O	Fm Hx of Carcinomas: NS; Surgical/Medical Complications: NS; Duration of Hospital Stay: NS
Brinksma et al. (21)	O	O	O	O	O	O	O	O	O	O	O	O	O	O	O
Broberger et al. (22)	O	O	O	O	O	O	NS	NS	O	O	NS	O	NS	O	O
Dabakuyo et al. (23)	O	S	O	S for sexual functioning only	O	O	S	O	O	O	O	O	O	O	O
Echteld et al. (24)	O	O	O	O	O	O	O	O	O	O	O	O	O	O	O
Gerlich et al. (25)	O	O	O	O	O	O	O	O	O	O	O	O	O	O	O
Hagedoorn et al. (26)	O	O	O	O	O	O	O	O	O	O	O	O	O	O	O
Hamidou et al. (27)	O	O	O	S (for some of Tx)	O	O	O	O	O	O	O	O	O	O	O
Hinz et al. (28)	O	NS	O	O	NS	O	NS	O	O	O	O	O	NS	O	O
Hinz (29)	O	O	O	O	NS	O	Vignette B: S	O	O	O	O	O	S	O	O
Ito et al. (30)	O	O	O	O	O	O	O	O	O	O	O	O	O	O	O
Jakola et al. (31)	O	O	O	O	O	O	O	O	O	Seizures: S (improved HQoL)	O	O	O	O	Mobility: NS; Self-care: NS; Usual Activities: NS
Jansen et al. (32)	O	O	O	O	O	O	O	O	O	Skin reactions: S; Low back pain—NS, Abdominal aches–NS, Loss of Hair–NS	O	O	O	O	O
Jorngarden et al. (33)	O	O	O	O	S	O	O	O	O	O	O	O	O	O	O
King-Kallimanis et al. (34)	NS	O	O	O	O	O	S	S	O	O	O	O	O	O	S
Korfage et al. (35)	O	O	O	O	O	O	O	O	O	O	O	O	O	O	O
Kvam et al. (14)	O	O	O	O	O	O	O	O	O	O	O	O	O	O	O
Oort et al. (44)	O	O	O	O	O	O	O	O	O	O	O	O	O	O	O
Ousmen et al. (37)	O	O	O	O	O	O	O	O	O	O	O	O	O	O	O
Rees et al. (38)	O	O	O	S	O	O	O	O	O	O	O	O	O	O	O
Salmon et al. (39)	O	NS	O	S	O	O	NS	O	O	O	NS	S	NS	O	Anxiety: NS; Optimism: NS
Sharpe et al. (13)	O	O	O	O	O	O	O	O	O	O	O	O	O	O	Life domain: S
Sharpley and Christie (40)	O	O	O	O	O	O	S	O	O	O	O	O	O	O	O
Sprangers et al. (17)	O	O	O	O	O	O	O	O	O	O	O	O	O	O	O
Tessier et al. (41)	O	O	O	O	O	O	NS	O	O	O	O	S	S	O	Financial status: S
Traa et al. (42)	NS	O	O	S	O	O	O	O	O	O	O	O	O	O	O
Verdam et al. (43)	O	O	O	O	O	O	O	O	O	O	O	O	O	O	O
Verdam et al. (44)	O	O	O	O	O	O	O	O	O	O	O	O	O	O	O
Visser et al. (45)	O	O	O	S	O	O	O	O	O	O	O	O	O	O	O
Visser et al. (46)	O	O	O	O	O	O	O	O	O	O	O	O	O	O	O
Visser et al. (4)	S	O	O	S	O	O	S	O	O	O	O	O	O	O	Social Comparisons: S
von Blackenburg et al. (47)	O	NS	O	NS	NS	O	O	S	O	NS	O	NS	NS	O	O
Westerman et al. (48)	O	O	O	O	O	O	O	O	O	O	O	O	O	O	O

*Dx, diagnosis; Fm Hx, family history; FSI, Fatigue Symptom Inventory; Tx, treatment; NS, not a statistically significant contributor; O, not assessed/considered in the model; S, statistically significant contributor*.

Table 3 displays demographics, perceived social support and clinical contributions of patient characteristics to main response shift statistical analyses. Of the 35 studies reviewed, three studies accounted for cancer type (4, 34, 42), five studies accounted for cancer stage (18, 23, 28, 39, 47), one study accounted for metastasis (20), 10 studies accounted for treatment type (4, 18, 20, 22, 27, 39, 41, 42, 45, 47), four studies accounted for time elapsed since diagnosis (survivorship time) (28, 29, 33, 47), two studies accounted for age at diagnosis (28, 33), 10 studies accounted for current age (4, 18, 22, 23, 28, 29, 34, 39–41), four studies accounted for sex (20, 22, 34, 47), three studies accounted for comorbidities other than the cancer diagnosis (31, 32, 47), two studies accounted for occupation (22, 39), three studies accounted for marital status (39, 41, 47), and seven studies accounted for education (18, 22, 28, 29, 39, 41, 47) in their main analyses. Cancer type (4), cancer stage (23), treatment type (4, 18, 23, 27, 39, 41, 42, 45), time elapsed since diagnosis (33), age at diagnosis (33), age at the time of testing (4, 18, 23, 29, 34, 40), sex (34, 47), marital status (39), comorbidity (31, 32), and education (29, 41) were factors that statistically significantly contributed to the main analyses. None of the studies reviewed reported information on the patients' race, or patients' perceived level of social support.

Table 4 indicates the presence or absence of a response shift, and the type of response shift design studied in the 35 studies reviewed. Statistical analyses and models also varied among these studies. Methods used to assess response shift in the studies reviewed included the then-test (21 studies of the 35) (4, 14, 17–23, 25, 27, 28, 30–32, 35, 37, 40, 41, 45, 46), the pre-test and post-test (12 studies) (13, 24, 26, 33, 34, 36, 41–44, 47, 48), with less frequent use of the anchoring/ideal scale (two studies) (23, 37), successive comparison (one study) (23), structural-equational modeling (one study) (39), or vignettes (two studies) (29, 35). The most common method for evaluating response shift that observed the emergence of a statistically significant response-shift effect was the then-test (19/21 studies). All studies reported at least one significant result for the variables examined in their model, with the exception of one study. Jakola et al. only examined global QoL, which was found to be not statistically significant (31). Table 4 displays effect sizes for the studies who reported them broken down by outcome measured. Overall effect sizes were mostly small to negligent and were reported in 16 studies (14, 18, 19, 21, 22, 24, 25, 28, 35–37, 39, 40, 43, 44, 46) four of which also reported moderate effect sizes (21, 22, 24, 46), and one additional study which revealed a large effect size (17). Across all types of cancer, 22 studies reported results for global QoL and statistically significant changes between evaluated time points were noted in 16 of these (13, 14, 19, 21–24, 26, 29, 32, 34–37, 44, 46), however only 10 of these studies reported effect sizes for their statistically significant effects. Of these, using Cohen's standard of interpretation, seven reported mostly negligible to small effect sizes <0.5 (14, 19, 35–37, 44, 46), and three reported medium effect sizes between ≥5 to <0.8 (largest medium effect size reported among the three studies was 0.7) (21, 22, 24), of which one involved children cancer patients (21). Among the 20 studies reporting results on physical functioning, 12 reported a statistically significant response shift (14, 19, 22, 25–27, 32, 35, 37, 42, 43, 46). Of these, five did not report effect sizes for their findings, six studies reported negligible to small (14, 19, 22, 25, 35, 37) and one reported a medium effect size (46). Response shift was also observed in six of the studies on role functioning (19, 25, 27, 36, 37, 46), five of which reported effect sizes; four rating negligible to small (19, 25, 36, 37, 46), and one of a medium effect size (46). Seven of the studies on emotional functioning (19, 23, 25–27, 37, 46) reported the presence of response shift, but only four reported effect sizes for their effect, of which all were small (19, 25, 37, 46). Eight studies examined response shift in cognitive functioning and found a significant response shift in four of those (23, 25, 27, 37), of which two reported effect sizes of small magnitude (25, 37). Of the five studies that found the presence of response shift sexual functioning three found a statistical significant response shift effect (19, 27, 35), two of which was of small effect size (19, 35). Social functioning was examined in 12 studies and evidence of response shift emerge in eight of them (19, 25, 27, 36, 37, 41, 42, 44), five of which had small effect sizes (19, 25, 36, 37, 44). Studies examining fatigue (17 studies) (14, 17–19, 22–24, 27, 29, 30, 32, 37, 39, 44–46, 48), pain (13 studies) (4, 14, 19, 24, 27, 29, 30, 34, 36, 37, 43, 44, 46), and a large portion of the studies that looked at mental health (14 studies) (28–30, 33–36, 40–44, 46, 47) which found a statistically significant effect had mostly small effect sizes. Specifically, of the eight studies reporting effect sizes for fatigue, seven had small effect sizes (14, 18, 19, 22, 24, 37, 39, 46) and one reported a large effect size (>0.9) (17). Of the six studies that reported effect sizes for their analyses on pain, five reported small effect sizes (14, 19, 36, 37, 46) and one reported a medium effect size (24). Four studies reported effect size for mental health assessment all of which were small (28, 35, 40, 44). Lastly, the presence of response shift was also noted in various other health related measures (14, 20, 23, 25, 27, 28, 32, 35–39, 41–44, 46, 47) and of the studies who reported these effects and reported effect sizes, they were mostly small to negligible (14, 19, 21, 22, 25, 28, 35–37, 39, 43, 44, 46).

**Table 4:** Response Shift indicators in studies assessing QoL among cancer patients and methods used to assess Response Shift.

**References**	**Cancer type**	**Methods used to assess response shift**	**Global QoL**	**Physical functioning**	**Role functioning**	**Emotional functioning**	**Cognitive functioning**	**Sexual functioning**	**Social functioning**	**Fatigue**	**Pain**	**MH**	**Other sig. results**
Andrykowski et al. (18)	Breast	Then-test	O	O	O	O	O	O	O	S (Average fatigue CT or RT: 0.35 SD Average fatigue CT + RT: 0.52 SD)	O	O	O
Anota et al. (19)	Breast	Then-test	S (at 3 months; −0.21)	S (at 6 months; 0.31)	S (at 3 and 6 months;−0.32, 0.30)	S (after hospitalization and 3 months; 0.21, 0.27)	NS	NS	S (at 3 and 6 months; −0.27, 0.22)	S (at 6 month; −0.43)	S (at 6 months; −0.23)	O	Nausea and vomiting- NS; Dyspnea—NS; Insomnia—S (at 3 and 6 months) −0.22, −0.18; Appetite loss—NS; Constipation—NS; Diarrhea—NS; Financial difficulties—NS; Body image—S (at 3 and 6 months) 0.48, 0.25; Future perspective—S (after hospitalization and 3 months) 0.24, 0.23; STSE—S (at 6 months) −0.24; Breast symptoms—S (at 6 months) −0.31; Arm symptoms—NS; Hair loss—NS
Bernhard et al. (20)	Colon	Then-test	NS—surgery	NS–surgery	O	O	O	O	O	O	O	O	S–subjective health
Brinksma et al. (21)	Hematologic; brain tumor; solid tumor	Then-test	S–for children (−0.74) and parents (−0.3)	NS	O	NS	NS	O	NS	O	O	O	O
Broberger et al. (22)	Lung	Then-test	S (3 months, 0.40; 6 months, 0.70)	S (3 months, 0.23)	O	O	O	O	O	S (3 months, 0.29)	O	O	O
Dabakuyo et al. (23)	Breast	Then-test, Ideal scale, Successive comparison scale	S	O	O	S	S	O	O	S	O	O	Insomnia—S, Appetite loss—NS, Diarrhea—NS, Future Perspectives—NS, Systemic therapy side effects—S
Echteld et al. (24)	Lung; colorectal: urogenital; breast; melanoma; sarcoma; other	Pre-test, post-test	S (0.60)	O	O	O	O	O	O	S (0.20)	S (0.66)	O	O
Gerlich et al. (25)	Prostate	Then-test	O	S (−0.44)	S (−0.47)	S (0.17)	S (−0.13)	O	S (−0.35)	O	O	O	O
Hagedoorn et al. (26)	Breast; gastrointestinal tumors; lymphomas; Genitourinary; lung; gynecological	Pre-test, post-test	S	S	O	S	O	O	O	O	O	O	O
Hamidou et al. (27)	Breast	Then-test	NS	S	S	S	S	S	S	S	S	O	Nausea—S, Dyspnea—S, Insomnia–S, Appetite Loss—S, Diarrhea–NS, Body Image–S, Future Perspectives–NS, Systemic Therapy Side Effects—S, Breast Symptoms–S, Arm Symptoms–S
Hinz et al. (28)	Prostate; kidney; bladder; testicles; penis; renal pelvis	Then-test	O	O	O	O	O	O	O	O	O	S (anxiety, 0.26; depression 0.30)	Distress–NS, Health dissatisfaction—S
Hinz (29)	Breast	Vignettes	Vignette A: S	NS	NS	NS	NS	NS	NS	NS	NS	NS	NS
Ito et al. (30)	Rectal	Then-test	NS	NS	NS	NS	O	NS	NS	NS	S	NS	O
Jakola et al. (31)	Glioma	Then-test	NS	O	O	O	O	O	O	O	O	O	O
Jansen et al. (32)	Breast	Then-test	S	S	O	O	O	O	O	S	O	O	Psychological well-being–NS
Jorngarden et al. (33)	CNS tumor; Ewing sarcoma; leukemia; lymphoma; osteosarcoma; other	Pre-test, post-test	O	O	O	O	O	O	O	O	O	S (Anxiety, depression, and mental health)	Vitality—S
King-Kallimanis et al. (34)	Lung; pancreas; esophageal; cervical	Pre-test, post-test with SEM models	S	NS	NS	NS	O	O	O	O	S	NS	Vitality- NS
Korfage et al. (35)	Prostate	Then-test, vignettes	S (7 month follow up, −0.43)	Urinary Leakage—S (1 month follow up, −0.32), Bowel Cramps—S (1 month follow up, −0.41; 7 month follow up, −0.39)	O	O	O	Erectile Dysfunction—S (1 month follow up, −0.57; 7 month follow up, −0.47)	O	O	O	S (7 month follow up, 0.17)	Vitality—S (1 month follow up, −0.28; 7 month follow up, −0.26),
Kvam et al. (14)	Multiple myeloma	Then-test	S (0.11)	S (−0.07)	O	O	O	O	O	S (0.25)	S (0.16)	O	O
Oort et al. (36)	Lung; pancreas; esophageal; cervical	Pre-test, post-test	S (0.14)	NS	S (0.27)	NS	O	O	S (−0.11)	O	S (0.30)	S	Vitality—S (0.00)
Ousmen et al. (37)	Breast	Then-test Anchor-base	3 months—S (0.20), 6 months–NS	3 months–NS, 6 months—S (−0.32)	3 months—S (0.32), 6 months—S (0–0.29)	3 months—S (−0.29), 6 months—NS	3 months—S (−0.19), 6 months—S (−0.15)	3 months—NS, 6 months—NS	3 months—S (0.28), 6 months—S (−0.23)	3 months—NS, 6 months—S (0.41)	3 months—NS, 6 months—S (0.23)	O	Nausea and vomiting—NS, Dyspnea—NS, Financial ^*^/difficulties–NS, Insomnia—S 3 months, 0.22 0, Appetite Loss—S (6 months, 0.19), Constipation—S (6 months, 0.16), Diarrhea—NS, Body image—S (0.37, −0.23), Sexual enjoyment—NS, Future perspectives: 3 months—S (−0.27), 6 months—NS, Systemic therapy side effects—NS, Breast
													symptoms—NS, Arm symptoms: 3 months—NS, 6 months—S (0.21)
Rees et al. (38)	Prostate	Then-test and pre-test, post-test	O	O	O	O	O	O	O	O	O	O	Urinary Function—S
Salmon et al. (39)	Breast	SEM modeling	O	O	O	O	O	O	O	Mental fatigue: S (0.27) Physical fatigue: S (0.41)	O	O	Activity reduction: S (0.59) Motivation reduction: S (0.43)
Sharpe et al. (13)	Multiple, but primarily lung and colorectal	Pre-test, post-test	S	O	O	O	O	O	O	O	O	O	O
Sharpley and Christie (13)	Breast	Then-test	O	O	O	O	O	O	O	O	O	S (anxiety, 0.25; depression 0.13)	O
Sprangers et al. (17)	Breas; lung; prostate; Hodgkin	Then-test	O	O	O	O	NS	O	O	S (0.94)	O	O	O
Tessier et al. (41)	Breast	Pre-test, post-test	O	NS	NS	NS	NS	O	S	O	O	O	NS—Life satisfaction
Traa et al. (42)	Colorectal	Pre-test, post-test	O	S	O	O	O	O	S	O	O	S	Environment—S
Verdam et al. (43)	Breast; prostate; lung; other	Pre-test, post-test	O	S	O	O	O	O	NS	–RS—NS	S	–RS—NS	Listlessness—NS; Sickness—S; Treatment related illness—NS
Verdam et al. (44)	Breast; colorectal; lung; other	Pre-test, post-test	S (−0.19)	NS	NS	NS	O	O	S (-0.05)	O	NS	S (0.08)	Vitality—S (−0.34), Health Comparison—S
Visser et al. (45)	Head and neck; gastrointestinal; gynecological; lung; breast; genitourinary tract; hematological malignancies; other	Then-test	O	O	O	O	O	O	O	S	O	O	O
Visser et al. (46)	Lung; periampullary; esophageal; cervical	Then-test, anchor recalibration	S (−0.15)	S (−0.58)	S (−0.53)	S (0.27)	O	O	NS	S (0.34)	S (−0.49)	S (0.39)	Vitality—S (−0.31)
Visser et al. (4)	Lung; periampullary; esophageal; cervical	Then-test	O	O	O	O	O	O	O	O	S	O	O
von Blackenburg et al. (47)	Breast; lung; urologic; digestive system; gynecologic; other	Pre-, post-test	NS	NS	O	O	O	O	O	O	O	S	S—life goals
Westerman et al. (48)	Lung	Pre-test, post-test	O	O	O	O	O	O	O	S	O	O	O

*Effect sizes in parentheses when provided by authors; direction of the response shift effect is indicated by a negative (underestimation) or a positive (overestimation) sign*.

*MH, mental health; NS, not a statistically significant contributor; O, not assessed/considered in the model; QoL, quality of life; RS, response shift; S, statistically significant contributor; Sig, significant; Tx, treatment*.

## Discussion

This study reviewed the presence and magnitude of response shift in studies assessing cancer patients' QoL over time. Evaluating the presence of response shift during the assessment of QoL measurement among cancer patients and its magnitude is important because it provides a measure of the extent to which the “true” effects of the cancer diagnosis and treatment can be masked by changes in the internal standard of measurement (otherwise assumed to be negligible) during these measurements. Undertaking a review for cancer, separate from that of other chronic conditions is important as QoL among cancer patients is known to be poorer compared to that of other non-cancer chronic conditions, some of which may predispose individuals to cancer (49).

Error in QoL measurement attributable to response shift could lead to failure to detect treatment toxicity and side-effects. This is important because when toxicity and side-effects are identified and acted upon through post-treatment interventions the result can lead to a reduction in their harmful effects on patients' quality of life, short and long term (2, 7). This review shows that response shift was present in 34 of the 35 studies assessed, although overall, the magnitude of the response shift found was negligible, to small, at best. The studies reviewed here showed large heterogeneity in the types of cancer assessed, patient characteristics and study designs. Among the 35 studies identified patients diagnosed with either breast (18 studies), lung (14 studies), prostate cancer (eight studies), and colorectal (eight studies) were the most commonly assessed populations.

All studies, with the exception of one (very small sample size) which was comprised of children, included older participants (mostly among 50–65 years old). Age, sex, time elapsed since diagnosis, and external factors were assessed in few studies (4, 10, 4, and 7, respectively) and, on average, half the time or less were not found to be statistically significantly contributing to the response shift-effect observed (2, 6, 1, and 5, respectively). The most common method used to assess and find a response-shift effect was the “then-test” (19/21 studies found a small albeit significant effect). Most studies that observed a statistically significant response shift effect had a time between assessments that varied between 3 and 6 months. However, there was significant heterogeneity between the baseline selected among the studies (Tables 1, 3) as some selected a post-diagnosis, pre-treatment baseline, others selected a post-treatment baseline, yet others arbitrarily chose a period of time that elapsed after treatment without controlling for the period of time that elapsed between diagnosis and baseline treatment in their analyses. All except one study (31) reported the presence of a statistically significant response shift in one or more QoL dimensions. In this one study, patients remained stable after surgery and were not stable simply due to response shift because none was observed (31). About a half (16 studies) of the 34 studies that found a statistically significant response shift and reported effect sizes for their results, revealed negligible effect sizes, indicating that overall response shift, while detectable it has a negligible influence on quality of life outcomes whether measured through validated and reliable questionnaires or self-reported answers to questions assessing QoL outcomes (14, 18, 19, 21, 22, 24, 25, 28, 35–37, 39, 40, 43, 44, 46). Among the different QoL subscales, the occurrence of a moderate effect size response shift were evident among four of these 16 studies which detected negligeable to small effect sizes, in the assessment of pain (24), physical limitations (46), global QoL (21, 22, 24), and social role functioning (46). One study only reported a large effect size response shift, for the assessment of fatigue (17). These results may suggest that response shift may be a phenomenon occurring particularly in measurement of physical aspects of functioning and possibly global QoL, although before any definitive conclusions are drawn, these results need to be replicated with larger sample sizes among homogeneous samples of cancer patients.

There are many reasons why most of the effect sizes reported in the studies we reviewed are small to negligible. One possibility could be the heterogeneity of length of time between their QoL assessments. Considering the possibility that response shift may take a short time to manifest or may become insignificant over time, it may be important to consider these variations when evaluating response shift. Indeed, of the six studies, of moderate, and one study, of large effect size, identified, all [except Broberger et al. (22)] assessed QoL at diagnosis or hospital admission and had a second assessment right after treatment suggesting that internal standards “shifted” within a narrow window of time from diagnosis to immediately post-treatment. Since this “shift,” when identified in other studies, was present but negligible in size, it may be possible that the “shift” may be strongest when assessed within a short time since diagnosis, preferably right after treatment. Future studies should assess if it is possible that response shift may be an artifact of chosen baseline assessment throughout the cancer journey timeline continuum, and length of time elapsed between diagnosis and post-treatment assessment, with longer periods of time leading to loss of strength in the effect identified. Baseline or pre-test assessments were given at different times between the 35 studies reviewed here. For example, baseline of pre-test assessments was administered sometimes at the time of diagnosis, 2-weeks after diagnosis, hospitalization, pre-surgery, right at the start of treatment, post-surgery, discharge, or were not specified. The time post-assessment also varied considerably, as each study selected different times of post-assessment. The majority of studies (16 studies) administered QoL assessment 3 months after baseline whereas nine studies measured 6 months after baseline. Most studies that observed a statistically significant response shift effect size were administered 3 months after baseline, with fewer observing an effect that was present at 6 months. Only four, of the 35, studies assessed the time elapsed between diagnosis and first/baseline testing and controlled for it in their analyses (28, 29, 33, 47). Future studies should consider controlling for this important factor in their analyses as this information has considerable relevance for quality of life outcomes due to expected consequences of specific treatments and for efforts to identify the expected rehabilitative needs of cancer survivors (50).

Furthermore, studies also varied in the length of time elapsed between longitudinal QoL assessments (e.g., 1 week, 3 months, 6 months) but also the number of time points assessed (19 studies measured QoL at multiple times, whereas 16 studies measured QoL only once after baseline assessment). Future studies should consider the inclusion of multiple time assessments to allow for the examination of the presence and strength of response shift over time.

A second reason why effect size, among the few studies reporting it, may have been very negligible, may be related to the heterogeneity of the samples, small number of participants in the samples examined and different methodologies adopted for testing for response shift. Few among the studies reviewed controlled for patient characteristics in their examination of response shift in heterogeneous samples (e.g., patients with various forms of cancer, of different ages, and different stages of cancer). None of the studies reviewed here evaluated the possible contribution of age at cancer diagnosis or race to response shift. Among the patient characteristics that were evaluated in the papers we identified, and reviewed, treatment type appeared to be the most influential contributor (found significant in eight of 10 studies that evaluated it) of response shift among cancer patients. None of the reviewed studies examined the contribution of perceived social support to response shift. Given that the relationship between social support and QoL is well-established (51–54), where social support is associated with improved QoL and is shown to influence the patients' level of perceived distress related to their cancer diagnosis, which in turn may alter their evaluation of their outcomes, future studies should consider controlling for its contribution to the presence or absence of response shift in patient reported outcomes (12, 51–54). Other important factors such as cancer type (found to be a statistically significant contributor in one of the three studies that reported it) and stage (a significant contributor in one of the five studies who examined it), the presence of comorbidities (found significant in two of the three studies who assessed it), occupation (evaluated in two studies), and marital status (found to be statistically significant in two of three studies who evaluated it) which have been documented to be associated with QoL outcomes among various cancer types, should be considered as possible confounds and included in future studies evaluating response shift given their considerable relevance to QoL outcomes among cancer patients due to efforts to identify modifiable and non-modifiable life factors in better survivorship (50). Lastly, we note the lack of standardization in the measurement and reporting of response shift in the studies reviewed here. Study designs in the 35 studies we reviewed included the “then” test (21 studies of the 35) and the “pre-test and post-test” (12 studies) methodology predominantly, with four other less adopted methodologies. Currently, there is still much debate surrounding the appropriate methodology for measuring response shift, and which statistical tests to use to analyze the data, which instruments accurately capture QoL, and what information should be recorded by researchers (4, 8, 31, 55–57). A standard method for collecting and reporting response shift data will aid the scientific community to justly determine whether the phenomenon of response shift exists, or if it is simply a methodological artifact.

While the presence of response shift of internal QoL standards among cancer patients may reduce the actual effect size of the QoL changes observed in longitudinal studies from one time point to another, the present review found small to negligible evidence to support its influence. An ideal methodology for assessing response shift in QoL measurement would be to include a time point assessment before diagnosis and compare it to post-diagnosis and post-treatment responses. Interestingly, one such assessment by Broberger et al., which was performed at 2–4 months before lung cancer diagnosis, found no decisive support for the hypothesis that a change in internal standards occurred in this group of patients (22). The explanation for the lack of response shift may be that patients would have adapted to the symptoms of their diagnosis at least to some extent prior to their diagnosis, or that the “shift” some studies observe may be part of the normal life fluctuations some people may experience rather than a consistent and stable phenomenon that is event bound (e.g., cancer diagnosis). Therefore, “response shift” may be capturing people's natural psychological adaptations to life circumstances which most generally eventually succumb to what we know as “regression to the mean” (58). This concept may be described as the process whereby changes in internal states may fluctuate, and go up or down depending on what life events an individual has to face from one given point in time to another, but that eventually they regress toward whatever may consist as the “average” response based on the internal states which generally define this individual (58).

Detecting unbiased or “real” cancer treatment effects is crucial not only to help fine-tune interventions, their administered length of time and intensity dosages, to inform patient education and empowerment programs in order to reduce negative side effects and improve patients' quality of life, but to also identify extreme (weather positive—resilience, or negative—extreme vulnerability) psychological adaptations to treatments that often challenge people's sense of identity. Since attrition in longitudinal studies may lead to loss of severely ill patients from the original sample, it, as opposed to response shift, may explain why QoL outcomes are relatively high in cancer patient groups reflecting the better health scores of the remaining group's members. Future studies using large sample sizes and better designed methodologies may contribute to a deeper understanding of whether response shift may be one of several factors influencing QoL assessments in light of changing life circumstances.

Given that the patient population samples in most studies reviewed here were heterogeneous with wide varieties of treatments, length of time between diagnosis and QoL assessments, treatment schedules, and cancer specific and demographic characteristics that were more often not accounted for in the analyses, response shift studies should be considered more in the hypothesis generating spectrum, until more studies are conducted to account for these limitations. The knowledge that a decrease in QoL outcomes post-treatment may be underestimated by a small amount should also be seen in light of the on-going discussion on the issue of clinically relevant changes (59).

It must be noted that this review is not without its limitations. First of all, the studies included in this systematic review were identified via electronic searches of three databases (MEDLINE, EMBASE, and PsychINFO) plus a manual review of the reference section of selected papers. It is possible that relevant articles pertaining to this review could have been missed using the aforementioned methods. Moreover, more than half of the studies in this review involved the then-test method, which is known to be susceptible to recall bias. For example, Litwin and McGuigan examined recall bias in men treated for prostate cancer and found inaccuracies in pre-treatment outcomes recall (56). Korfage et al. also argue that the use of general health QoL measures (e.g., SF-36, EQ-5D, EORTC-C30) may not be ideal for accurately measuring patient-reported health because the generic measures may not include questions on important disease-specific side effects (60). For example, sexual, urinary and/or bowel dysfunctions experienced by prostate cancer survivors post-treatment are not well-captured by generic general health QoL measures, although specific measures of these conditions (e.g., IPSS- The International Prostate Symptom Score) are successful at capturing poor QoL in these domains in this population (55–57, 60). Donohoe also hypothesizes that high levels of social support may lead to better adaptation to the cancer diagnosis and its side-effects, which would present itself as a response shift. These external factors are often not taken into consideration because many of the general health QoL measures do not have questions assessing them (12). Therefore, response shift studies that use generic measures may reflect the measures used rather than accurate changes in perceived outcomes. Thus, given the large number of then-test studies in this review, the results should be interpreted within caution (8). Lastly, aggregating the studies we reviewed to compute a pooled effect size was not possible given the heterogeneity of study designs and measures included in this review. Lastly, this review on response shift focused solely on cancer patient samples, which limit its generalizability to other chronically ill patient populations. Thus, future studies are needed to replicate these effects with larger sample sizes while controlling for possible sample characteristics confounds before these results should be considered generalizable. At the time of this review, the clinical significance of response shift on QoL outcome measurements was still being elucidated, with inconsistent findings stemming from individual studies and an indefinite conclusion being reported by a previous meta-analysis (7) and the current review.

## Author Contributions

GI, RR, JB, and LM: conception. GI, RR, and JB: methodology. JB, AI, LM, CB, RG, TL, ZL, and GI: studies search. AI, JB, GI, TL, ZL, RG, and LM: results. RR, GI, JB, and AI: discussion. GI, JB, AI, LM, RG, RR, ZL, and TL: manuscript draft preparation. GI, RR, JB, AI, and LM: edits.

### Conflict of Interest Statement

The authors declare that the research was conducted in the absence of any commercial or financial relationships that could be construed as a potential conflict of interest.
