# Supplementary material for: The Role of Response-Shift in Studies Assessing Quality of Life Outcomes Among Cancer Patients: A Systematic Review
Source: Front Oncol. 2019 Aug 20;9:783. doi: 10.3389/fonc.2019.00783 (PMC6710367; doi:10.3389/fonc.2019.00783)
Supplement: Supplementary file 1 [file Table_1.DOCX]

To include in Appendix

| Cohort Studies |  |  |  |  |  |  |  |  |  |
| --- | --- | --- | --- | --- | --- | --- | --- | --- | --- |
| Author | Study type | Selection 1 | Selection 2 | Selection 3 | Selection 4 | Comparability | Outcome 1 | Outcome 2 | Outcome 3 |
| Andrykowski et al., 2009 | Cohort |  |  | * | * | * |  | * | * |
| Anota et al., 2014 | Cohort | * | * | * | * | * |  | * | * |
| Bernhard et al., 2001 | Cohort | * |  | * | * | * |  | * |  |
| Blackenburg et al., 2014 | Cohort |  |  | * | * | * |  | * | * |
| Brinksma et al., 2014 | Cohort |  |  | * | * | * |  | * | * |
| Broberger et al., 2006 | Cohort |  |  | * | * | * |  | * | * |
| Dabakuyo et al., 2013 | Cohort | * |  | * | * | * |  |  | * |
| Echteld et al., 2005 | Cohort |  |  | * | * | * |  | * |  |
| Gerlich et al., 2016 | Cohort |  |  | * | * | * |  | * | * |
| Hagedoorn et al., 2002 | Cohort |  |  | * | * | * |  | * | * |
| Hamidou et al., 2014 | Cohort | * |  | * | * | * |  | * | * |
| Ito et al., 2010 | Cohort |  |  | * | * | * |  | * | * |
| Jakola et al., 2017 | Cohort |  |  | * | * | * |  |  | * |
| Jansen et al., 2000 | Cohort |  |  | * | * | * |  | * |  |
| King-Kallimanis et al., 2009 | Cohort | * |  | * | * | * |  |  | * |
| Korfage et al., 2007 | Cohort |  |  | * | * | * |  | * | * |
| Kvam et al., 2010 | Cohort | * |  | * | * | * |  | * | * |
| Oort et al., 2005 | Cohort |  |  | * | * | * |  | * |  |
| Ousmen et al., 2016 | Cohort | * |  | * | * | * |  | * |  |
| Salmon et al., 2017 |  | * |  | * | * | * |  | * |  |
| Sharpe et al., 2005 | Cohort |  |  | * | * | * |  | * |  |
| Sharpley, & Christie, 2007 | Cohort | * |  | * | * | * |  |  |  |
| Sprangers et al., 1999 | Cohort |  |  | * | * | * |  | * | * |
| Tessier et al., 2017 | Cohort |  |  | * | * | * |  | * | * |
| Traa et al., 2015 | Cohort |  |  | * | * | * |  | * | * |
| Verdam et al., 2016 | Cohort |  |  | * | * | * |  | * | * |
| Verdam et al., 2015 | Cohort | * |  | * | * | * |  | * |  |
| Visser et al., 2005 | Cohort | * |  |  | * | * |  | * |  |
| Visser et al., 2013 | Cohort | * |  |  | * | * |  | * |  |
| Visser et al., 2000 | Cohort | * |  | * | * | * |  | * | * |
| Westerman et al., 2007 | Cohort |  |  | * | * | * |  | * | * |

| Case Control Studies |  |  |  |  |  |  |  |  |  |
| --- | --- | --- | --- | --- | --- | --- | --- | --- | --- |
| Author | Study type | Selection 1 | Selection 2 | Selection 3 | Selection 4 | Comparability | Exposure 1 | Exposure 2 | Exposure 3 |
| Hinz et al., 2011 | Case-control | * | * | * |  | * | * | * | * |
| Hinz et al., 2017 | Case-control | * | * | * | * | * | * |  |  |
| Jorngarden et al., 2007 | Case-control | * | * | * | * | * | * | * |  |
| Rees et al., 2003 | Case-control | * | * |  | * | * | * |  |  |
