# Supplementary material for: The Role of Response-Shift in Studies Assessing Quality of Life Outcomes Among Cancer Patients: A Systematic Review
Source: Front Oncol. 2019 Aug 20;9:783. doi: 10.3389/fonc.2019.00783 (PMC6710367; doi:10.3389/fonc.2019.00783)
Supplement: Supplementary file 2 [file Table_2.docx]

Appendix 1

# MEDLINE Search Strategy

*Ovid MEDLINE(R) Epub Ahead of Print, In-Process & Other Non-Indexed Citations, Ovid MEDLINE(R) Daily and Ovid MEDLINE(R)*

1. exp Neoplasms/

2. cancer*.tw.

3. carcinoma*.tw.

4. malignan*.tw.

5. tumor*.tw.

6. neoplas*.tw.

7. adeno*.tw.

8. metasta*.tw.

9. 1 or 2 or 3 or 4 or 5 or 6 or 7 or 8

10. (response adj (shift* or change*)).tw.

11. recalibrat*.tw.

12. (reprioritiz* or reprioritis*).tw.

13. (reconceptualiz* or reconceptualis*).tw.

14. 10 or 11 or 12 or 13

15. 9 and 14
